# The prognostic value of the pan-immune-inflammation value in cancer patients treated with immune checkpoint inhibitors: a systematic review and meta-analysis

**DOI:** 10.3389/fonc.2026.1841286

**Published:** 2026-08-14

**Authors:** Le Yang, Ziqian Zhao, Munawar Anwar, Jiawei Chen, Yuquan Dai, Yeliya Yeerboli, Zuqiang Xu, Zizhang Wang, Meihui Shan, Chao Dong

**Affiliations:** 1 The Clinical Medical Research Center of Breast and Thyroid Tumor in Xinjiang, Tumor Hospital Affiliated to Xinjiang Medical University, Urumqi, China; 2 Xinjiang Key Laboratory of Oncology, The Third Affiliated Teaching Hospital (Affiliated Cancer Hospital) of Xinjiang Medical University, Urumqi, China; 3 Clinical Medicine Department of Xinjiang Medical University, Urumqi, China

**Keywords:** immunotherapy, meta-analysis, pan-immune inflammation value, prognosis, tumor

## Abstract

**Objective:**

Immune checkpoint inhibitors (ICIs) show substantial therapeutic potential in advanced or metastatic solid malignancies, yet optimization of clinical benefit remains an unmet need. This systematic review and meta-analysis evaluates the pretreatment pan-immune-inflammation value (PIV) as a prognostic biomarker in ICI-treated solid tumors.

**Methods:**

We conducted comprehensive searches in PubMed, EMBASE, Cochrane Library and Web of Science covering all records available up to January 2026. Hazard ratios (HRs) with 95% confidence intervals (CIs) were retrieved, and pooled analyses were performed using STATA 18.0 to quantify correlations between PIV and survival results. Overall survival (OS) was considered the primary outcome, with disease-free, progression-free, and recurrence-free survival (DFS/PFS/RFS) as secondary endpoints.

**Results:**

Twenty-two studies comprising 28 cohorts and 3,272 participants were incorporated. Multivariate meta-analysis detected that an elevated PIV was related to shorter OS (HR = 2.31; 95% CI: 2.02–2.65; P < 0.00001) and inferior DFS/PFS/RFS (HR = 1.79; 95% CI: 1.42–2.26; P < 0.00001). Subgroup analyses denoted that follow-up duration, geographic region, PIV cutoff definitions, and cancer type may have modified the predictive performance of PIV for DFS/PFS/RFS.

**Conclusions:**

Among patients receiving ICIs, an elevated pretreatment PIV may serve as a potentially useful adverse prognostic biomarker for survival outcomes.

**Systematic Review Registration:**

https://www.crd.york.ac.uk/PROSPERO/, identifier CRD420261290575.

## 1 Introduction

Cancer constitutes one of the most pressing transnational health challenges, with approximately 20 million incident cases and 9.7 million deaths reported annually (1). Conventional anticancer approaches, including chemotherapy and radiotherapy, are constrained by limited tumor specificity, suboptimal intratumoral drug penetration, a persistent risk of recurrence, and substantial systemic toxicities (2, 3). These limitations have driven the development and clinical adoption of cancer immunotherapy as a transformative therapeutic strategy. Immune checkpoint inhibitors are antibody therapeutics targeting PD-1/PD-L1 or CTLA-4, thereby attenuating suppressive signaling that restrains T-cell priming and restoring antitumor immunity (4–6).

Although durable clinical benefit has been achieved with immune checkpoint inhibitors (ICIs) in selected patients, therapeutic responses remain limited for the majority. Objective response rates (ORRs) have been reported to reach 40–70% in melanoma and Hodgkin lymphoma, yet are typically only 10–25% across most other malignancies (7–11). Therefore, reliable biomarkers are required to stratify individuals who are most likely to respond favorably to immunotherapy. improve the precision of response and outcome prediction, and enable more individualized treatment strategies.

Systemic inflammation is intrinsically linked to tumor initiation and progression, while dysregulated energy metabolism constitutes a recognized hallmark of malignancy (12, 13). Consequently, the PIV, a novel biomarker reflecting immune-inflammatory status, is emerging as a pivotal indicator for the prediction of immunotherapy efficacy. Previous investigations have indicated that elevated PIV is strongly linked to inferior clinical outcomes in individuals receiving ICI therapy (14–17). For instance, in a single-center study involving 129 participants with metastatic melanoma, it was demonstrated by Mesti et al. that elevated pretreatment PIV predicted compromised OS and significantly shortened PFS and RFS over a two-year follow-up period (18). Similarly, a multicenter retrospective analysis undertaken by Lien et al. studied 192 patients with recurrent or metastatic head and neck squamous cell carcinoma receiving pembrolizumab or nivolumab between 2018 and 2022; the findings revealed that elevated baseline PIV was also prognostic for diminished OS in this cohort (19). Nevertheless, the comprehensive clinical utility of PIV in patients receiving ICI-centered cancer treatment remains an area necessitating further exploration.

Notably, a meta-analysis was previously undertaken by Kuang et al. (20), which incorporated seven studies published between 2021 and 2023. Although it was reported that elevated PIV correlated with inferior OS and PFS in individuals receiving ICIs, the scope of that analysis was restricted to literature available prior to 2023. Since that time, a substantial volume of clinical data has emerged, yielding findings that have not been entirely consistent. Consequently, the present study is designed to expand upon the prior analysis by integrating 15 recently published investigations, with the objective of comprehensively validating the prognostic utility of PIV in ICI-treated solid malignancies.

## 2 Materials and methods

### 2.1 Search strategy

This meta-analysis followed the PRISMA statement (21). The study protocol was recorded in the International Prospective Register of Systematic Reviews (PROSPERO: CRD420261290575). Two assessors worked separately to construct the retrieval protocol (LY and ZQZ). A comprehensive systematic search was performed on January 10, 2026 using four sources: PubMed, Embase, Web of Science, and the Cochrane Library. No language or regional restrictions were imposed during the screening and retrieval steps to ensure no includable report was missed. In PubMed, the search strategy is constructed using the following terms: “Immune Checkpoint Inhibitors” [MeSH], “PD-L1 inhibitors,” “PD-1 inhibitors,” “CTLA-4 inhibitors,” “nivolumab,” “tremelimumab,” “cemiplimab,” “pembrolizumab,” “PIV,” and “pan-immune-inflammation value.” **Supplementary Table 1** details the search approach.

### 2.2 Study selection

Study eligibility was determined using the PICOS criteria. Only reports involving malignant-disease cohorts managed with ICIs were considered, reported the PIV, and provided at least one survival endpoint (OS or DFS/PFS/RFS). In addition, studies were required to report HRs with 95% CIs and P values, and to stratify patients into high- and low-PIV groups using a prespecified cutoff. Studies were discarded if they involved hematologic malignancies, were reviews, case reports, or letters, or did not report HRs for OS or DFS/PFS/RFS. Study selection was performed independently by two reviewers, and the results were subsequently consolidated.

### 2.3 Data extraction

Two investigators independently collected the data with a prespecified Excel form, and the collected records were consolidated by a third investigator. Any discrepancies were settled through consensus after discussion. The following information was collected: first author, publication year, region, study design, sample size, patient age, cancer type, study period, ICI regimen, follow-up duration, TNM stage, PIV cutoff, timing of PIV assessment, statistical model, and HRs with 95% CIs for OS and DFS/PFS/RFS. PIV was defined as neutrophil count × platelet count × monocyte count/lymphocyte count, and the cutoff value used for PIV stratification in each included study was extracted.

### 2.4 Quality assessment

Study quality was appraised with the Newcastle–Ottawa Scale (NOS), which evaluates observational studies in three components: selection, comparability, and outcome. Two studies received the maximum NOS score of 9 points (14, 19). Two reviewers separately rated methodological rigor, and any discordance was reconciled through consensus after discussion.

### 2.5 Statistical analysis

HRs for OS and DFS/PFS/RFS, together with 95% CIs, were obtained from each included study. Data for analysis were categorized into unadjusted (univariable) and adjusted (multivariable) sets. Unadjusted estimates were those reported without controlling for potential confounders, whereas adjusted estimates were obtained from models accounting for multiple confounding factors. Pooled effect estimates were obtained using fixed-or random-effects framework depending on heterogeneity across reports. Heterogeneity was quantified with Cochran’s Q test and the Higgins I² statistic (22). Substantial heterogeneity was defined as I² > 50% or P < 0.05. A fixed effects approach was chosen when heterogeneity was not notable; otherwise, a random-effects approach was adopted. Potential drivers of heterogeneity were explored using subgroup analyses. Robustness was examined using leave-one-out sensitivity analyses. Publication bias was inspected by funnel plots and Egger’s test; when significant asymmetry was identified, trim-and-fill analyses were conducted to obtain bias-adjusted effects. Additionally, For every endpoint, a four-tier confidence rating (high,moderate, low, or very low) was assigned under GRADE to support the final conclusions (23). All tests were two-sided, and P < 0.05 was considered statistically significant. Statistical analyses were performed using STATA (version 18.0) and Review Manager (version 5.4).

## 3 Results

### 3.1 Study selection and characteristics

**Figure 1:**
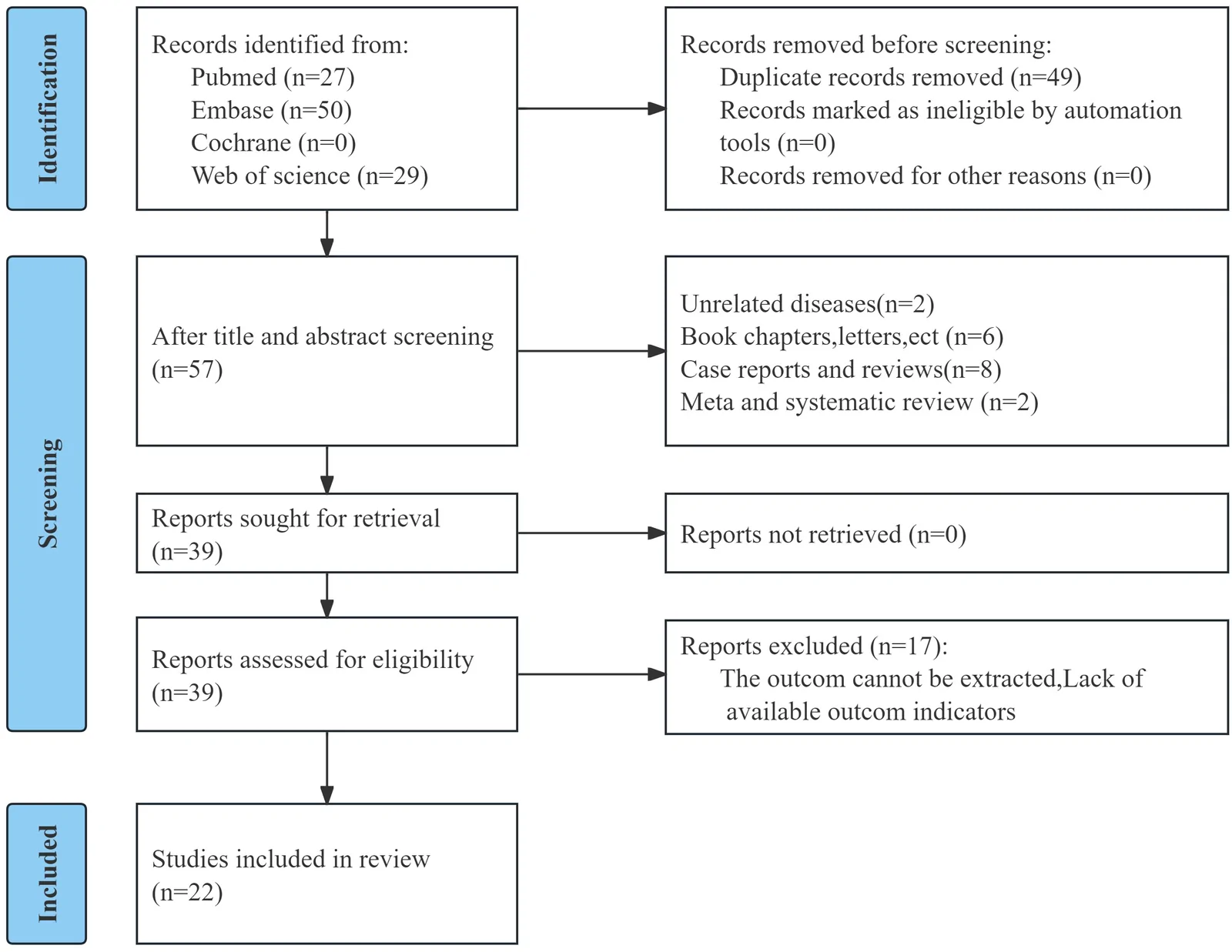
Flow chart of literature screening.

The preliminary search yielded 106 references in total; after duplicate removal, 57 citations remained. After screening the titles and abstracts, 18 records were removed. Subsequently, from 39 reports carried forward to in-depth review, 17 did not meet inclusion requirements and were removed. Ultimately, 22 publications representing 3,272 participants were integrated into the meta-analysis (**Figure 1**) (14, 15, 18, 19, 24–41). From these 22 studies, which were published between 2021 and 2025, 28 separate cohorts were extracted. The majority of the cohorts originated from China (n=13), followed by multicenter collaborations (n=9). The most prevalent malignant neoplasms were lung cancer (11 cohorts) and gastrointestinal cancer (8 cohorts). Regarding study design, 27 cohorts were retrospective, while the remaining one was prospective. All included studies were published in English, with study periods ranging from 2012 to 2024. Across these cohorts, the median patient age extended from 55 to 70 years, and the PIV cutoff values varied between 120 and 1,312. The principal features of the eligible studies are presented in **Table 1**.

**Table 1:** Basic characteristics of the included literature.

Author	Study period	Region	Study design	Cancer type	ICI treatment	Timing of detection	No. of patients	Gender (male/female)	Median follow-up (months)	Mean/median Age	TNM stage	PIV cut-off	Outcomes
Wang, F. 2024 (38)	2018-2023	Multicenter	Retrospective cohort	Biliary Tract Cancer(BTC)	Anti-PD-(L)1 antibodies	Pre-treatment	118	73/45	NA	55.00	NA	209.27	OS
Li, Y.Q. 2025a (30)	2017-2023	China	Retrospective cohort	Non-Small Cell Lung Cancer(NSCLC)	ICIs	within 7 days before treatment	102	82/20	13.80	61.60	III-IV	341.62	PFS
Li, Y.Q. 2025b	2017-2023	China	Retrospective cohort	Non-Small Cell Lung Cancer(NSCLC)	ICIs	within 7 days before the 3rd treatment cycle	102	82/20	13.80	61.60	III-IV	329.49	PFS
Li, Y.Q. 2025c	2017-2023	China	Retrospective cohort	Non-Small Cell Lung Cancer(NSCLC)	ICIs	within 7 days before the 5th treatment cycle	102	82/20	13.80	61.60	III-IV	238.49	PFS
Su, Z. 2024 (36)	2018-2022	China	Retrospective cohort	Recurrent or Metastatic Head and Neck Cancer(R/M HNC)	ICIs	Pre-treatment	57	50/7	12.00	56.00	NA	940.27	OS, PFS
Mesti, T. 2023a (18)	2018-2020	Slovenia	Retrospective cohort	Metastatic Melanoma	ICIs	Pre-treatment	129	84/53	22.50	66.20	NA	390.00	OS, PFS
Mesti, T. 2023b (18)	2018-2020	Slovenia	Retrospective cohort	Metastatic Melanoma	ICIs	before the second cycle of ICI	129	84/53	22.50	66.20	NA	390.00	OS, PFS
Ulas, A. 2024 (37)	2019-2024	Turkey	Retrospective cohort	Advanced Non-Small Cell Lung Cancer(aNSCLC)	nivolumab	Pre-treatment	104	94/10	22.00	62.00	IIIB/IV	682.90	OS, PFS
Ma, S.X. 2024 (32)	2019-2022	China	Retrospective cohort	Advanced Non-Small Cell Lung Cancer(aNSCLC)	Anti-PD-(L)1 antibodies	Pre-treatment	161	127/34	NA	65 ± 8.7	IIIB/IV	250.51	OS
Lien, M.Y. 2024 (19)	2018-2022	Multicenter	Retrospective cohort	Recurrent or Metastatic Head and Neck Cancer(R/M HNC)	pembrolizumab or nivolumab	Pre-treatment	192	176/16	16.30	58.00	I-IV	966.00	OS, PFS
Chen, Y.Q. 2023 (14)	2019-2022	China	Retrospective cohort	Advanced Non-Small Cell Lung Cancer(aNSCLC)	pembrolizumab or nivolumab	Pre-treatment	269	232/37	NA	67.00	III-IV	387.00	OS, PFS
Sooi, K. 2022 (35)	2015-2022	Singapore	Retrospective cohort	Advanced Non-Small Cell Lung Cancer(aNSCLC)	ICIs	Pre-treatment	203	142/61	NA	63.00	NA	790.00	OS, PFS
Corti, F. 2021 (25)	2014-2020	Multicenter	Retrospective cohort	Metastatic Colorectal Cancer(mCRC)	ICIs	Pre-treatment	163	90/73	31.00	70.00	NA	492.00	OS, PFS
Cheng, X.Y. 2025a (24)	2019-2021	Multicenter	Retrospective cohort	Esophageal Squamous Cell Carcinoma(ESCC)	Anti-PD-(L)1 antibodies	During the course of treatment	86	66/20	40.40	58.00	I-IV	813.10	PFS
Cheng, X.Y. 2025b	2019-2021	Multicenter	Retrospective cohort	Esophageal Squamous Cell Carcinoma(ESCC)	Anti-PD-(L)1 antibodies	Pre-treatment	86	66/20	40.40	58.00	I-IV	316.70	PFS
Cheng, X.Y. 2025c	2019-2021	Multicenter	Retrospective cohort	Esophageal Squamous Cell Carcinoma(ESCC)	Anti-PD-(L)1 antibodies	Post-treatment	86	66/20	40.40	58.00	I-IV	259.90	PFS
Guven, D.C. 2021 (28)	2016-2020	Turkey	Retrospective cohort	advanced cancer	Anti-PD-(L)1 antibodies	Pre-treatment	120	86/34	9.62	61.00	NA	513.40	OS, PFS
Zeng, R. 2021a (41)	2015-2021	China	Retrospective cohort	Small Cell Lung Cancer(SCLC)	Atezolizumab	Pre-treatment	53	34/19	NA	65.00	NA	581.95	OS, PFS
Zeng, R. 2021b	2015-2021	China	Retrospective cohort	Small Cell Lung Cancer(SCLC)	Anti-PD-(L)1 antibodies	Pre-treatment	84	75/9	NA	65.00	NA	581.95	OS, PFS
Lei, W.Q. 2024 (29)	2019-2023	China	Retrospective cohort	Advanced Non-Small Cell Lung Cancer(aNSCLC)	ICIs	Pre-treatment	108	83/25	NA	60.00	VI	297.49	PFS
Qi, W.X. 2023 (34)	2019-2022	Multicenter	Prospective cohort	Esophageal Squamous Cell Carcinoma(ESCC)	pembrolizumab	Pre-treatment	51	44/7	20.00	62.00	II-IVA	232.83	PFS
Feng, J. 2024 (26)	2019-2022	China	Retrospective cohort	Esophageal Squamous Cell Carcinoma(ESCC)	NICT	Pre-treatment	218	197/21	19.00	63.3 ± 6.7	II-IVA	373.60	OS, DFS
Liang, X.X. 2024 (31)	2014-2021	China	Retrospective cohort	Hepatocellular Carcinoma(HCC)	ICIs	Pre-treatment	265	227/38	20.00	65.00	NA	120.00	OS, RFS
Midik, M.M. 2025 (33)	2012-2023	Turkey	Retrospective cohort	cancer	ICIs	Pre-treatment	226	143/83	55.60	56.50	II-IV	1312.00	OS, PFS
Fu, M. 2024 (15)	2018-2023	China	Retrospective cohort	Advanced Gastric Cancer(aGC)	Anti-PD-(L)1 antibodies	Pre-treatment	84	54/30	NA	59.24 ± 11.59	IV	499.08	OS, PFS
Gao, Z. 2025 (27)	2020-2024	Multicenter	Retrospective cohort	Metastatic Colorectal Cancer(mCRC)	bevacizumab and anti-PD-1 immunotherapy	Pre-treatment	29	21/8	13.30	60.00	IV	NA	PFS
Yekeduz, E. 2022 (40)	NA	Multicenter	Retrospective cohort	Metastatic Renal Cell Carcinoma(mRCC)	nivolumab	Pre-treatment	152	117/35	29.10	60.00	NA	372.00	OS, PFS
Yang, S. 2025 (39)	2019-2024	China	Retrospective cohort	Advanced Non-Small Cell Lung Cancer(aNSCLC)	ICIs	Pre-treatment	298	273/25	42.11	64.00	IIIB–IVB	208.00	OS

### 3.2 Methodological quality

All included publications exhibited Newcastle-Ottawa Scale (NOS) scores ≥6, suggesting overall good methodological rigor across the selected literature (**Supplementary Table 1**).

### 3.3 Meta-analysis results

#### 3.3.1 PIV and unadjusted OS (univariate analysis)

**Figure 2:**
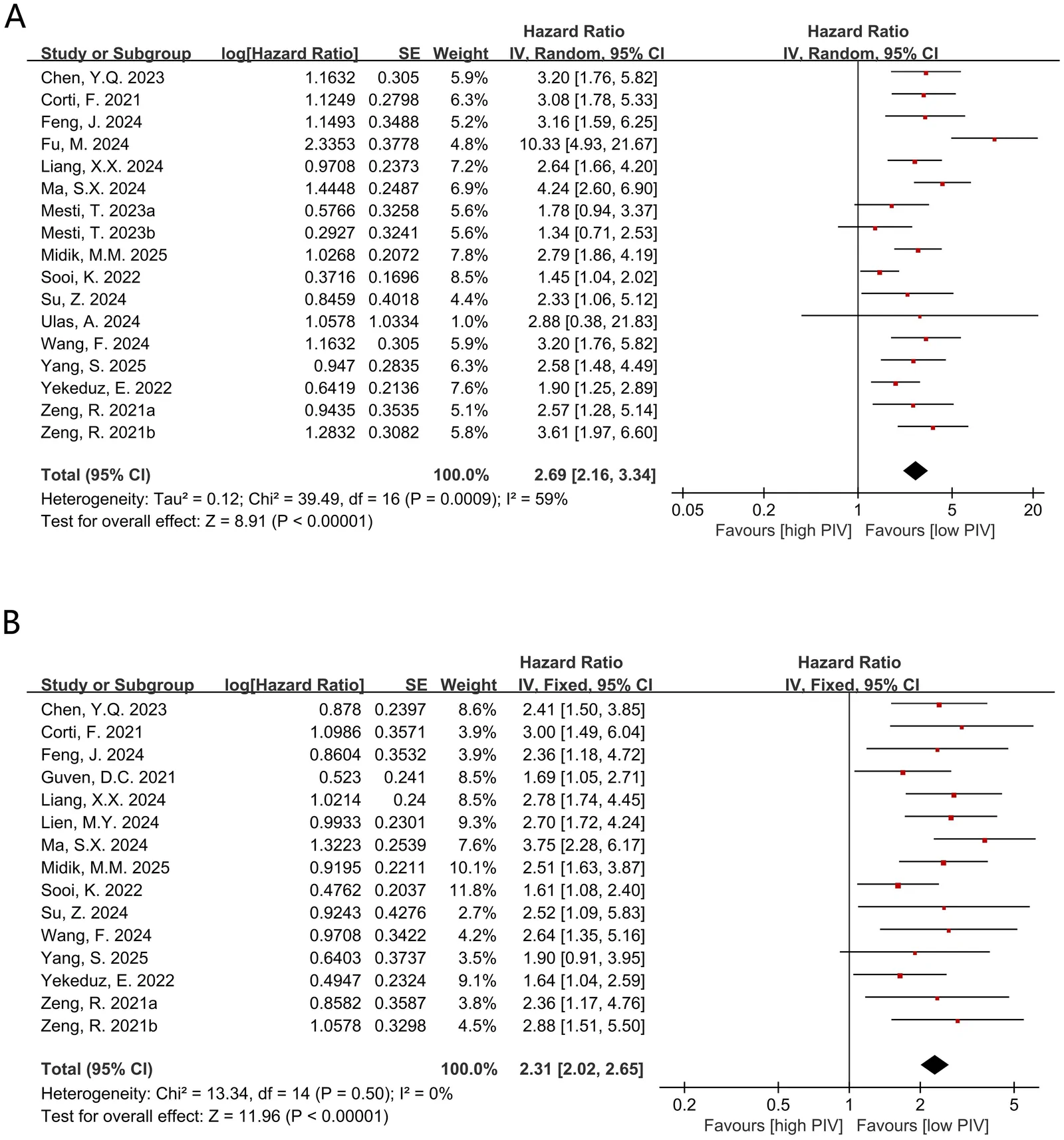
Forest plot that demonstrates the comparison between high pan-immune-inflammation value (PIV) score and low PIV score for predicting the overall survival (OS) of cancer patients based on univariate **(A)** and multivariate **(B)** data.

Unadjusted overall survival (OS) was reported in 17 comparisons among patients with cancer treated with ICIs and stratified by baseline PIV. An elevated pretreatment PIV was linked to worse OS compared with a low PIV (HR, 2.69; 95% CI, 2.16–3.34; P<0.00001) (**Figure 2A**). Considerable heterogeneity was noted (I²=59%; P = 0.0009); accordingly, a random-effects model was adopted. To investigate explanatory factors of heterogeneity, subgroup analyses were undertaken and showed a consistent association across prespecified subgroups (**Table 2**). Exploratory analyses further suggested that follow-up duration, age, geographic region, PIV cutoff definition, and cancer type contributed to between-study heterogeneity.

**Table 2:** Summary of meta-analysis and subgroup analysis results for univariate and multivariate overall survival (OS) and disease-free, progression-free, and recurrence-free survival (DFS/PFS/RFS) outcomes. HR, hazard ratio; DFS, disease-free survival; PFS, progression-free survival; RFS, recurrence-free survival; OS, overall survival; PIV, pan-immune-inflammation value.

Subgroup	OS(univariate analysis)				OS(multivariate analysis)				DFS/PFS/RFS(univariate analysis)				DFS/PFS/RFS(multivariate analysis)			
	Study group	HR [95%CI]	*P* value	*I* ^2^	Study group	HR [95%CI]	*P* value	*I* ^2^	Study group	HR [95%CI]	*P* value	*I* ^2^	Study group	HR [95%CI]	*P* value	*I* ^2^
Total	17	2.69 [2.16, 3.34]	<0.00001	59%	15	2.31 [2.02, 2.65]	<0.00001	0%	23	1.71 [1.41, 2.09]	<0.00001	80%	15	1.79 [1.42, 2.26]	<0.00001	66%
Sample size																
<200	11	2.86 [2.09, 3.92]	<0.00001	62%	9	2.41 [2.00, 2.91]	<0.00001	11%	18	1.65 [1.29, 2.11]	<0.0001	78%	12	1.76 [1.27, 2.43]	0.0007	73%
≥200	6	2.43 [1.82, 3.25]	<0.00001	53%	6	2.21 [1.80, 2.70]	<0.00001	0%	5	1.83 [1.55, 2.15]	<0.00001	0%	3	1.80 [1.47, 2.20]	<0.00001	0%
Follow-up																
<25 months	6	2.20 [1.68, 2.89]	<0.00001	0%	5	2.36[1.86, 3.00]	<0.00001	0%	11	1.44 [1.09, 1.89]	0.009	71%	8	1.55 [0.99, 2.43]	0.05	82%
≥25 months	4	2.49 [1.97, 3.14]	<0.00001	0%	4	2.14 [1.64, 2.80]	<0.00001	0%	6	1.62 [1.21, 2.17]	0.001	48%	3	1.77 [1.30, 2.41]	0.0003	0%
Mean/median Age																
<65	9	2.73 [1.92, 3.88]	<0.00001	70%	9	2.05 [1.72, 2.44]	<0.00001	0%	16	1.69 [1.31, 2.17]	<0.0001	63%	9	1.74 [1.15, 2.62]	0.009	78%
≥65	8	2.73 [2.11, 3.52]	<0.00001	36%	6	2.85 [2.27,3.57]	<0.00001	0%	7	1.75 [1.28, 2.40]	0.0005	86%	6	1.78 [1.49, 2.13]	<0.00001	0%
Region																
China	9	3.37 [2.60, 4.37]	<0.00001	39%	8	2.67 [2.17, 3.30]	<0.00001	0%	11	1.97 [1.54, 2.51]	<0.00001	61%	9	1.75 [1.43, 2.14]	<0.00001	22%
Other	5	1.82 [1.30, 2.55]	0.0005	44%	3	1.89 [1.47, 2.42]	<0.00001	19%	5	1.42 [1.05, 1.91]	0.02	68%	2	1.24 [0.88, 1.75]	0.21	0%
Multicenter	3	2.52 [1.77, 3.60]	<0.00001	30%	4	2.30 [1.76, 3.01]	<0.00001	10%	7	1.51 [0.96, 2.38]	0.07	69%	4	2.48 [1.33, 4.66]	0.005	83%
PIV cut-off																
<350	4	3.11 [2.40, 4.04]	<0.00001	0%	4	2.86 [2.16, 3.79]	<0.00001	0%	8	1.29 [0.96, 1.73]	0.09	55%	5	1.47 [0.94, 2.30]	0.09	57%
≥350	13	2.57 [1.96, 3.38]	<0.00001	64%	11	2.16 [1.85, 2.53]	<0.00001	0%	14	1.96 [1.51, 2.55]	<0.00001	84%	10	1.93 [1.45, 2.57]	<0.00001	71%
Cancer type																
Lung cancer	7	2.72 [1.87, 3.95]	<0.00001	64%	6	2.32 [1.87, 2.88]	<0.00001	34%	9	1.73 [1.38, 2.16]	<0.00001	40%	7	1.66 [1.23, 2.22]	0.0008	39%
Gastrointestinal cancer	3	4.52 [2.17, 9.44]	<0.0001	74%	2	2.66 [1.63, 4.35]	<0.0001	0%	8	2.01 [1.17, 3.45]	0.01	78%	3	1.92 [1.36, 2.71]	0.0002	0%
Hepatopancreatobiliary Cancer	2	2.84 [1.97, 4.10]	<0.00001	0%	2	2.73 [1.86, 4.01]	<0.00001	0%	1	1.83 [1.30, 2.56]	0.0005	NA	1	1.96 [1.39, 2.75]	0.0001	NA
Other	5	2.07 [1.61, 2.64]	<0.00001	8%	5	2.13 [1.71, 2.65]	<0.00001	0%	5	1.42[1.08, 1.86]	0.01	67%	4	1.87 [0.92, 3.81]	0.08	90%

#### 3.3.2 PIV and adjusted OS (multivariate analysis)

Adjusted overall survival (OS) was reported in 15 comparisons among ICI-treated patients stratified by PIV. Pooled estimates revealed that a raised PIV was connected with decreased OS (HR, 2.31; 95% CI, 2.02–2.65; P<0.00001) (**Figure 2B**). No considerable heterogeneity was identified (I²=0%; P = 0.50); therefore, a fixed-effects model was adopted. Subgroup analyses further indicated that this association remained consistent across predefined strata, as summarized in **Table 2**.

#### 3.3.3 PIV and unadjusted DFS/PFS/RFS (univariate analysis)

**Figure 3:**
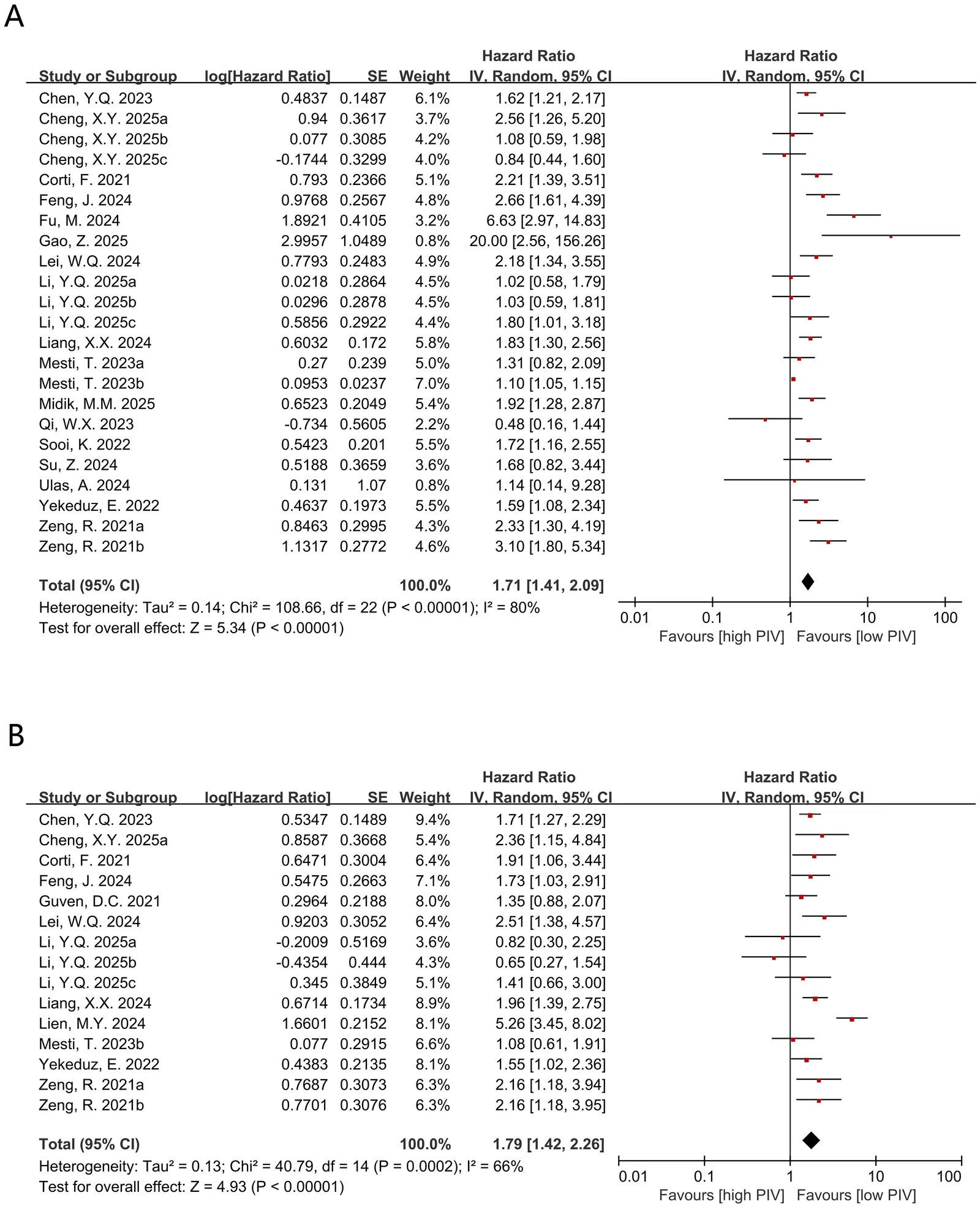
Forest plot that demonstrates the comparison between high pan-immune-inflammation value (PIV) score and low PIV score for predicting the disease-free, progression-free, and recurrence-free survival (DFS/PFS/RFS) of cancer patients based on univariate **(A)** and multivariate **(B)** data.

Unadjusted DFS/PFS/RFS was evaluated in 23 comparisons among individuals with malignancy receiving ICIs and stratified by PIV. The pooled analysis showed that a higher PIV was associated with unfavorable DFS/PFS/RFS (HR, 1.71; 95% CI, 1.41–2.09; P<0.00001) (**Figure 3A**). Marked between-study heterogeneity was noted (I²=80%; P<0.00001); therefore, a random-effects model was implemented. To clarify possible contributors of heterogeneity, subgroup analyses were undertaken (**Table 2**). The overall association was largely preserved across subgroups; however, prognostic significance was not observed in the multicenter setting subgroup (P = 0.05) or in studies using a PIV cutoff<350 (P = 0.09), whereas significant associations were retained in the remaining strata. Exploratory analyses further suggested that sample size, follow-up duration, and cancer type contributed to heterogeneity across studies.

#### 3.3.4 PIV and adjusted DFS/PFS/RFS (multivariate analysis)

Data on PIV and DFS/PFS/RFS were available from 15 comparisons. Using a random-effects model, a high PIV was correlated with inferior DFS/PFS/RFS (HR, 1.79; 95% CI, 1.42-2.26; P<0.00001) (**Figure 3B**). Subgroup analyses indicated that prognostic significance was not observed in studies with follow-up < 25 months (P = 0.05), those conducted in other regions (P = 0.21), those using a cutoff<350 (P = 0.09), or those involving other cancer types (P = 0.08), whereas significant associations were retained in the remaining strata. Exploratory analyses further suggested that sample size, follow-up duration, median age, cancer type, and geographic region contributed to between-study heterogeneity (**Table 2**).

### 3.4 Sensitivity analysis

**Figure 4:**
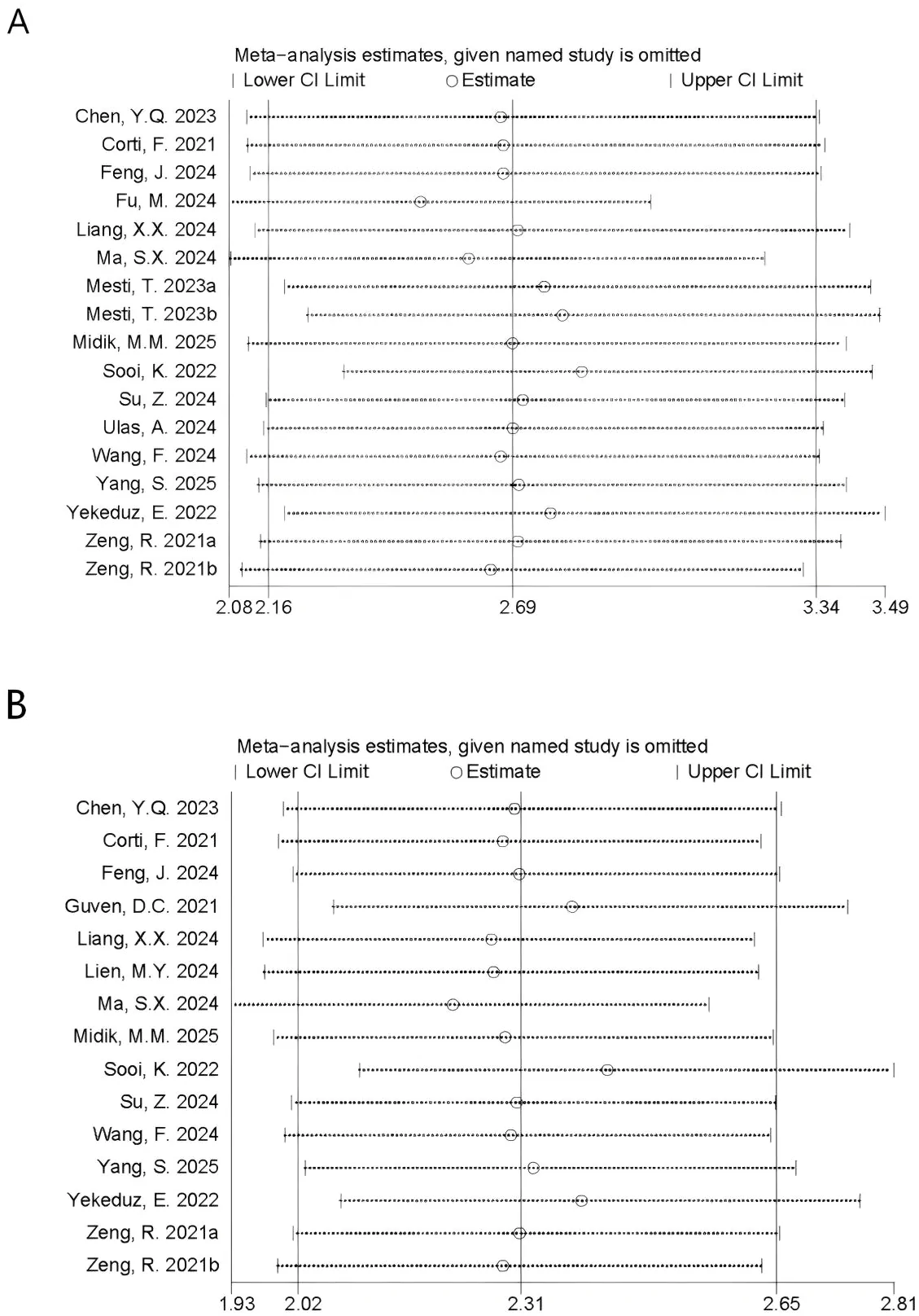
Sensitivity analysis of overall survival (OS) based on univariate **(A)** and multivariate **(B)** data.

**Figure 5:**
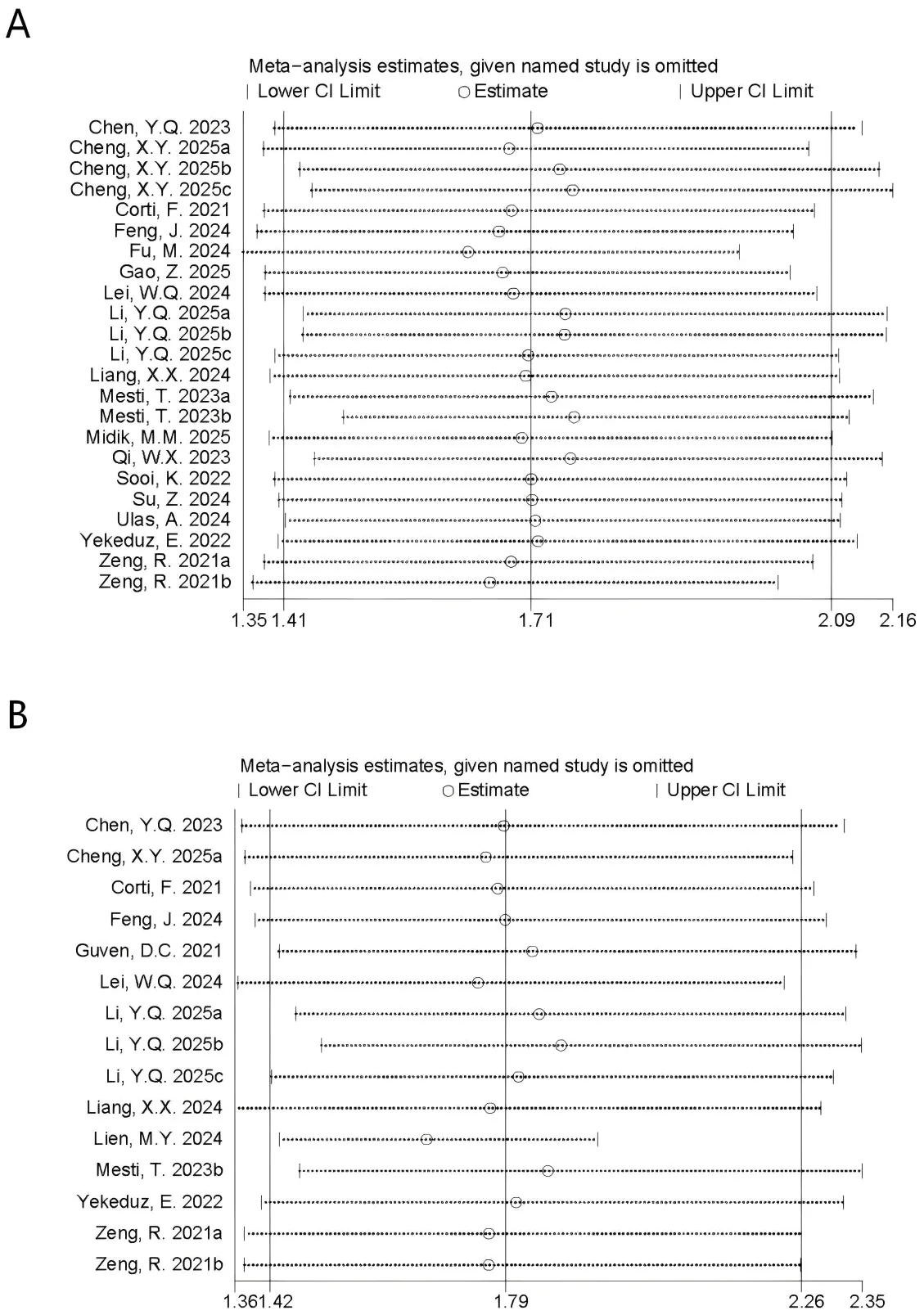
Sensitivity analysis of disease-free, progression-free, and recurrence-free survival (DFS/PFS/RFS) based on univariate **(A)** and multivariate **(B)** data.

Sensitivity analyses were implemented to appraise the stability of the pooled estimates. In both univariable and multivariable datasets, leave-one-out analyses indicated that the pooled HRs with 95% CIs for OS (**Figures 4A, B**) and DFS/PFS/RFS (**Figures 5A, B**) remained statistically significant after sequential removal of each publication.

### 3.5 Publication bias

**Figure 6:**
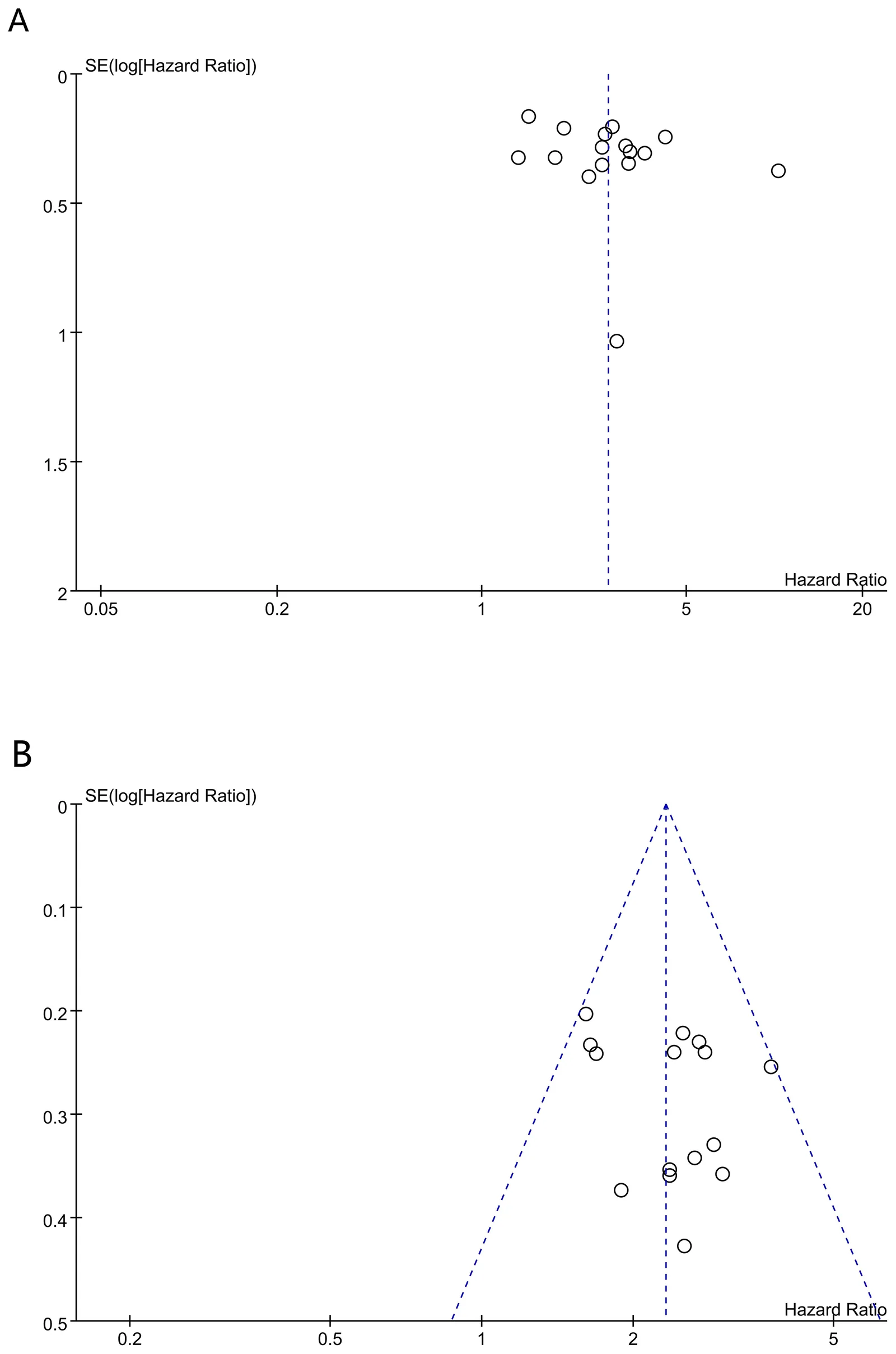
Funnel plot for the evaluation of publication bias for overall survival (OS) based on univariate **(A)** and multivariate **(B)** data.

**Figure 7:**
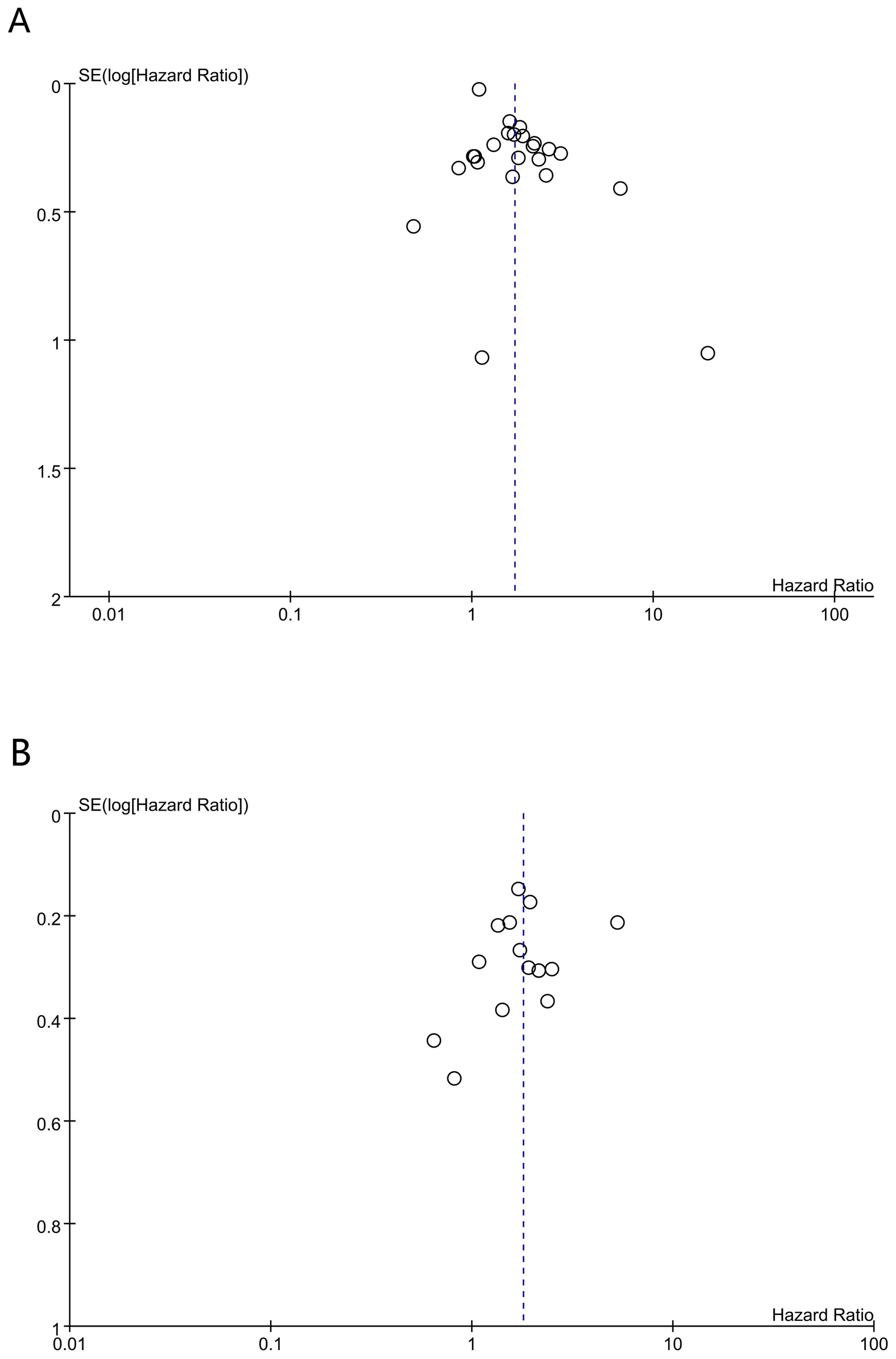
Funnel plot for the evaluation of publication bias for disease-free, progression-free, and recurrence-free survival (DFS/PFS/RFS) based on univariate **(A)** and multivariate **(B)** data.

Publication bias was appraised using funnel plots and Egger’s regression test. In both univariable (unadjusted) and multivariable (adjusted) datasets, Egger’s test did not demonstrate publication bias for unadjusted OS (p=0.116), adjusted OS (p=0.308), or adjusted DFS/PFS/RFS (p=0.371). Conversely, indication of publication bias was detected for the association between PIV and unadjusted DFS/PFS/RFS (p<0.00001). Accordingly, the trim-and-fill approach was utilized to examine how publication bias might influence the pooled results.No clearly missing studies were estimated by the procedure; therefore, no imputation was performed. After trim-and-fill adjustment, the pooled effect estimate for DFS/PFS/RFS remained essentially unchanged (HR, 1.71; 95% CI, 1.41-2.09; p<0.00001) (**Supplementary Figure 1**), implying that the observed correlation between elevated PIV and inferior DFS/PFS/RFS was robust and unlikely to have been materially driven by publication bias. Consistently, funnel plots appeared symmetric for unadjusted OS (**Figure 6A**), adjusted OS (**Figure 6B**), and adjusted DFS/PFS/RFS (**Figure 7A**), whereas asymmetry was observed for unadjusted DFS/PFS/RFS (**Figure 7B**).

### 3.6 GRADE assessment

Evidence certainty for each pooled outcome was evaluated with the GRADE. The evidence was judged to be low quality for unadjusted OS, moderate quality for adjusted OS, and very low quality for both unadjusted and adjusted DFS/PFS/RFS. Detailed GRADE ratings were provided in **Table 3**.

**Table 3:** GRADE rating of each outcome.

No. of study groups	Outcomes	HR/OR	95%CI	*I*^2^; P value	Risk of bias	Inconsistency	Indirectness	Imprecision	Publication bias	Plausible confounding	Magnitude of effect	Dose-response gradient	GRADE
17	OS (univariate analysis)	2.69	2.16, 3.34	59%; P = 0.0009	No serious risk	Serious inconsistency	No serious indirectness	No serious imprecision	Undetected	Would not reduce effect	YES	No	Low
15	OS (multivariate analysis)	2.31	2.02, 2.65	0%; P = 0.50	No serious risk	No serious inconsistency	No serious indirectness	No serious imprecision	Undetected	Would not reduce effect	YES	No	Moderate
23	DFS/PFS/RFS (univariate analysis)	1.71	1.41, 2.09	80%; P <0.00001	No serious risk	Serious inconsistency	No serious indirectness	No serious imprecision	Strongly suspected	Would not reduce effect	No	No	Very low
15	DFS/PFS/RFS (multivariate analysis)	1.79	1.42, 2.26	66%; P = 0.0002	No serious risk	Serious inconsistency	No serious indirectness	No serious imprecision	Undetected	Would not reduce effect	No	No	Very low

## 4 Discussion

Across 22 studies comprising 3,272 patients, the prognostic utility of PIV in patients with malignant neoplasms receiving ICIs was supported. An elevated PIV was associated with decreased OS and DFS/PFS/RFS. However, the pooled DFS/PFS/RFS result should be interpreted as a broad disease-control-related survival outcome rather than as evidence specific to any single endpoint. These associations remained consistent in both univariable and multivariable analyses, indicating that the observed relationship was not materially attenuated after adjustment for measured confounders. Taken together, PIV may function as an adverse prognostic marker among individuals receiving ICIs and may help refine risk stratification and clinical decision-making in this setting, although further prospective validation is warranted.

In comparison with the prior meta-analysis by Kuang et al. (20), several differences were observed. Kuang et al. reported that raised PIV was correlated with poorer OS and PFS compared with lower PIV, whereas the present analysis additionally demonstrated prognostic value for DFS/RFS. This study provides a more comprehensive synthesis of the available evidence for ICI-treated cancers (20). First, 22 studies were included, substantially more than the 7 studies analyzed by Kuang et al., thereby strengthening the evidentiary basis. Second, subgroup analyses were performed to stratify OS and DFS/PFS/RFS according to geographic region, sample size, PIV cutoff, mean age, cancer type, follow-up duration, and analytic model, which helps identify settings in which PIV is most informative and clarifies potential sources of heterogeneity. Finally, the pooled estimates were analyzed separately as unadjusted (univariable) and adjusted (multivariable) effects, a separation that had not been made in the meta-analysis by Kuang et al. Data from univariable and multivariable analyses are crucial for a thorough understanding of PIV’s predictive ability for the prognosis of individuals with malignancies.

Subgroup analyses further characterized heterogeneity in the prognostic performance of PIV among ICI-treated patients and suggested potential effect modifiers. In region-stratified analyses, meaningful connection between PIV and prognosis was identified in researches undertaken outside China or multicenter cohorts. This lack of statistical significance is plausibly owing to the paucity of studies within these strata and may also reflect population-level differences in genetic background, dietary patterns, access to healthcare, and tumor biology. Therefore, larger, internationally representative studies are needed, and standardized protocols for PIV assessment are required to determine whether prognostic performance is consistent across geographic settings. Regarding the analysis of PIV cutoff thresholds, it was found that a threshold greater than 350 demonstrated superior predictive value compared to a threshold less than 350. This observation suggests that future predictive models might benefit from establishing a PIV cutoff exceeding 350. Alternatively, optimal thresholds could be determined through comprehensive adjustments based on patient specific covariates, such as tumor stage, therapeutic efficacy, treatment response, and age.

Although substantial clinical evidence has supported high PIV as an adverse prognostic factor, the mechanisms linking PIV to outcomes in malignancy remain incompletely understood. Biological plausibility is provided by the four components of PIV: Lymphocytes are central to the control of immune responses, able to kill and inhibit the formation and spread of tumor cells (42). Cancer cells frequently exploit immune-escape strategies to suppress lymphocyte activity, which in turn facilitates tumor growth and metastatic spread (43). Immune-cell infiltration within malignant tissue has been linked to survival outcomes and treatment efficacy under immune-based therapies (44). In addition, recent studies show that platelets are involved in diverse signaling networks that regulate antitumor immunity and disease progression (45); it is reported that elevated platelet counts after pretreatment are related to inferior survival outcomes among patients with malignancy (46–48). On the other hand, at the biological mechanism level, neutrophils are considered key regulators of tumor progression. They create favorable conditions for tumor metastasis by releasing multiple inflammatory factors such as matrix metalloproteinase-9, neutrophil elastase, and interleukin-8 (49). Finally, peripheral blood monocytes are the main source of M2-type tumor-associated macrophages in the tumor microenvironment. These M2-type macrophages differentiated from circulating monocytes can induce an immune suppression state, thereby promoting tumor metastasis (50). Previous evidence shows that the increase of absolute monocyte count often suggests shortened survival of patients, which is closely related to its immune suppression characteristics.

Limitations Several limitations inherent to this meta-analysis warrant acknowledgment. First, considerable between-study heterogeneity was detected among the eligible studies, particularly within the analysis of PIV regarding DFS/PFS/RFS. Moreover, although DFS, PFS, and RFS were combined to increase the available evidence for secondary survival outcomes, these endpoints are not clinically identical; therefore, the pooled DFS/PFS/RFS findings should be interpreted cautiously. This heterogeneity may impair the reliability and generalizability of the pooled effect estimates. Although heterogeneity was explored through subgroup and sensitivity analyses, residual confounding could not be fully eliminated because study populations and clinical contexts differed with respect to baseline characteristics, molecular tumor subtypes, and neoadjuvant regimens. This limitation is particularly relevant because most included studies were retrospective, which increases susceptibility to selection and measurement biases; moreover, key clinical variables were often incompletely reported, constraining mechanistic interpretation and clinical translation. PIV, a composite was calculated from peripheral neutrophil, platelet, monocyte, and lymphocyte counts, is also vulnerable to distortion by intercurrent infection, comorbid conditions, concomitant medications, and inter-laboratory measurement variability-factors that are frequently unmeasured or insufficiently adjusted for in the source studies. In addition, PIV cutoff values were not standardized among researches, and the absence of an established threshold reduces between-study comparability and may weaken consistency of inference. Finally, although the DFS/PFS/RFS association remained robust after trim-and-fill procedures, Egger’s test suggested potential publication bias, implying that negative or null findings may be underreported and therefore underrepresented in the evidence base.

Large-scale, prospective multicenter investigations are needed to verify the prognostic utility of PIV in patients undergoing ICIs. Standardization of the timing of PIV assessment, calculation methods, and cutoff definitions is required, and comprehensive capture of treatment details, complications, and concomitant medications should be ensured to improve interpretability and reproducibility. In addition, investigation of clinically actionable strategies (such as immunomodulatory approaches and nutritional support) to favorably modify PIV and potentially improve outcomes remains an important direction for future research.

## 5 Conclusion

Overall, this meta-analysis suggested that a raised PIV was associated with adverse OS and DFS/PFS/RFS among individuals receiving ICIs. However, because most included studies were retrospective and the DFS/PFS/RFS analyses showed substantial heterogeneity with low or very low GRADE certainty, these findings should be interpreted with caution. These observations underscore PIV as a potentially effective prognostic biomarker that provides clinically relevant risk information in ICI-treated cancers. Future work should validate its prognostic performance in broader, prospectively enrolled multicenter cohorts and evaluate whether integration with established clinical and molecular biomarkers improves risk stratification and supports more individualized treatment strategies.

## Data Availability

The original contributions presented in the study are included in the article/**Supplementary Material**. Further inquiries can be directed to the corresponding author/s.
