# Supplementary material for: The prognostic value of the pan-immune-inflammation value in cancer patients treated with immune checkpoint inhibitors: a systematic review and meta-analysis
Source: Front Oncol. 2026 Aug 14;16:1841286. doi: 10.3389/fonc.2026.1841286 (PMC13521758; doi:10.3389/fonc.2026.1841286)
Supplement: Supplementary file 1 [file Table1.docx]

| Supplementary Table S1. Detailed search strategy in four databases. | |
| --- | --- |
| Database | Search strategy |
| Pubmed | (("Immune Checkpoint Inhibitors"[Mesh]) OR ((((((((((((((((((((((((((((((((((((((((Camrelizumab) OR (Sintilimab)) OR (Tislelizumab)) OR (Toripalimab)) OR (Envafolimab)) OR (Immune Checkpoint Inhibitors)) OR (Immune Checkpoint Inhibitor)) OR (Immune Checkpoint Blockers)) OR (Immune Checkpoint Blockade)) OR (Immune Checkpoint Inhibition)) OR (PD-L1 Inhibitors)) OR (PD L1 Inhibitors)) OR (PD-L1 Inhibitor)) OR (PD L1 Inhibitor)) OR (Programmed Death-Ligand 1 Inhibitors)) OR (Programmed Death Ligand 1 Inhibitors)) OR (PD-1-PD-L1 Blockade)) OR (PD 1 PD L1 Blockade)) OR (CTLA-4 Inhibitors)) OR (CTLA 4 Inhibitors)) OR (CTLA-4 Inhibitor)) OR (CTLA 4 Inhibitor)) OR (Cytotoxic T-Lymphocyte-Associated Protein 4 Inhibitors)) OR (Cytotoxic T Lymphocyte Associated Protein 4 Inhibitors)) OR (Cytotoxic T-Lymphocyte-Associated Protein 4 Inhibitor)) OR (Cytotoxic T Lymphocyte Associated Protein 4 Inhibitor)) OR (PD-1 Inhibitors)) OR (PD-1 Inhibitor)) OR (PD 1 Inhibitors)) OR (PD 1 Inhibitor)) OR (Programmed Cell Death Protein 1 Inhibitor)) OR (Programmed Cell Death Protein 1 Inhibitors)) OR (Pembrolizumab)) OR (Nivolumab)) OR (Atezolizumab)) OR (Ipilimumab)) OR (Avelumab)) OR (Tremelimumab)) OR (Durvalumab)) OR (Cemiplimab))) AND (((pan-immune-inflammation-value) OR (pan-Immune-inflammatory value)) OR (PIV)) |
| Embase | ((Immune Checkpoint Inhibitors or (Camrelizumab or Sintilimab or Tislelizumab or Toripalimab or Envafolimab or Immune Checkpoint Inhibitors or Immune Checkpoint Inhibitor or Immune Checkpoint Blockers or Immune Checkpoint Blockade or Immune Checkpoint Inhibition or PD-L1 Inhibitors or PD L1 Inhibitors or PD-L1 Inhibitor or PD L1 Inhibitor or Programmed Death-Ligand 1 Inhibitors or Programmed Death Ligand 1 Inhibitors or PD-1-PD-L1 Blockade or PD 1 PD L1 Blockade or CTLA-4 Inhibitors or CTLA 4 Inhibitors or CTLA-4 Inhibitor or CTLA 4 Inhibitor or Cytotoxic T-Lymphocyte-Associated Protein 4 Inhibitors or Cytotoxic T Lymphocyte Associated Protein 4 Inhibitors or Cytotoxic T-Lymphocyte-Associated Protein 4 Inhibitor or Cytotoxic T Lymphocyte Associated Protein 4 Inhibitor or PD-1 Inhibitors or PD-1 Inhibitor or PD 1 Inhibitors or PD 1 Inhibitor or Programmed Cell Death Protein 1 Inhibitor or Programmed Cell Death Protein 1 Inhibitors or Pembrolizumab or Nivolumab or Atezolizumab or Ipilimumab or Avelumab or Tremelimumab or Durvalumab or Cemiplimab)) and (pan-immune-inflammation-value or pan-Immune-inflammatory value or PIV)).af. |
| Web of Science | ((Immune Checkpoint Inhibitors) OR ((((((((((((((((((((((((((((((((((((((((Camrelizumab) OR (Sintilimab)) OR (Tislelizumab)) OR (Toripalimab)) OR (Envafolimab)) OR (Immune Checkpoint Inhibitors)) OR (Immune Checkpoint Inhibitor)) OR (Immune Checkpoint Blockers)) OR (Immune Checkpoint Blockade)) OR (Immune Checkpoint Inhibition)) OR (PD-L1 Inhibitors)) OR (PD L1 Inhibitors)) OR (PD-L1 Inhibitor)) OR (PD L1 Inhibitor)) OR (Programmed Death-Ligand 1 Inhibitors)) OR (Programmed Death Ligand 1 Inhibitors)) OR (PD-1-PD-L1 Blockade)) OR (PD 1 PD L1 Blockade)) OR (CTLA-4 Inhibitors)) OR (CTLA 4 Inhibitors)) OR (CTLA-4 Inhibitor)) OR (CTLA 4 Inhibitor)) OR (Cytotoxic T-Lymphocyte-Associated Protein 4 Inhibitors)) OR (Cytotoxic T Lymphocyte Associated Protein 4 Inhibitors)) OR (Cytotoxic T-Lymphocyte-Associated Protein 4 Inhibitor)) OR (Cytotoxic T Lymphocyte Associated Protein 4 Inhibitor)) OR (PD-1 Inhibitors)) OR (PD-1 Inhibitor)) OR (PD 1 Inhibitors)) OR (PD 1 Inhibitor)) OR (Programmed Cell Death Protein 1 Inhibitor)) OR (Programmed Cell Death Protein 1 Inhibitors)) OR (Pembrolizumab)) OR (Nivolumab)) OR (Atezolizumab)) OR (Ipilimumab)) OR (Avelumab)) OR (Tremelimumab)) OR (Durvalumab)) OR (Cemiplimab))) AND (((pan-immune-inflammation-value) OR (pan-Immune-inflammatory value)) OR (PIV)) (Topic) |
| Chochrane | ((Immune Checkpoint Inhibitors or (Camrelizumab or Sintilimab or Tislelizumab or Toripalimab or Envafolimab or Immune Checkpoint Inhibitors or Immune Checkpoint Inhibitor or Immune Checkpoint Blockers or Immune Checkpoint Blockade or Immune Checkpoint Inhibition or PD-L1 Inhibitors or PD L1 Inhibitors or PD-L1 Inhibitor or PD L1 Inhibitor or Programmed Death-Ligand 1 Inhibitors or Programmed Death Ligand 1 Inhibitors or PD-1-PD-L1 Blockade or PD 1 PD L1 Blockade or CTLA-4 Inhibitors or CTLA 4 Inhibitors or CTLA-4 Inhibitor or CTLA 4 Inhibitor or Cytotoxic T-Lymphocyte-Associated Protein 4 Inhibitors or Cytotoxic T Lymphocyte Associated Protein 4 Inhibitors or Cytotoxic T-Lymphocyte-Associated Protein 4 Inhibitor or Cytotoxic T Lymphocyte Associated Protein 4 Inhibitor or PD-1 Inhibitors or PD-1 Inhibitor or PD 1 Inhibitors or PD 1 Inhibitor or Programmed Cell Death Protein 1 Inhibitor or Programmed Cell Death Protein 1 Inhibitors or Pembrolizumab or Nivolumab or Atezolizumab or Ipilimumab or Avelumab or Tremelimumab or Durvalumab or Cemiplimab)) and (pan-immune-inflammation-value or pan-Immune-inflammatory value or PIV)).af. |
