# Supplementary material for: The prognostic value of the pan-immune-inflammation value in cancer patients treated with immune checkpoint inhibitors: a systematic review and meta-analysis
Source: Front Oncol. 2026 Aug 14;16:1841286. doi: 10.3389/fonc.2026.1841286 (PMC13521758; doi:10.3389/fonc.2026.1841286)
Supplement: Supplementary file 2 [file Table2.docx]

| Supplementary Table S2. Quality evaluation of the eligible studies with Newcastle–Ottawa scale. | | | | | | | | | |
| --- | --- | --- | --- | --- | --- | --- | --- | --- | --- |
| Study | Selection | | | | Comparability | | Outcome | | |
|  | Representative-ness | Selection of  non-exposed | Ascertainment  of exposure | Outcome not present at start | Comparability on most important factors | Comparability on other risk factors | Assessment of outcome | Long enough follow-up (median≥1 year) | Adequacy  (completeness) of follow-up |
| Wang, F. et al. | * | * | * | * | - | - | * | * | * |
| Li, Y.Q. et al. | * | * | * | * | - | - | * | * | * |
| Su, Z. et al. | * | * | * | * | - | - | * | * | * |
| Mesti, T. et al. | * | * | * | * | - | - | * | * | * |
| Ulas, A. et al. | * | * | * | * | - | - | * | * | * |
| Ma, S.X. et al. | * | * | * | * | * | - | * | * | * |
| Lien, M.Y. Z et al. | * | * | * | * | * | * | * | * | * |
| Chen, Y.Q. et al. | * | * | * | * | * | * | * | * | * |
| Sooi, K. et al. | * | * | * | * | - | - | * | * | * |
| Corti, F. et al. | * | * | * | * | - | - | * | * | * |
| Cheng, X.Y. et al. | * | - | * | * | - | - | * | * | * |
| Guven, D.C. et al. | * | * | * | * | - | - | * | * | * |
| Zeng, R. et al. | * | - | * | * | - | * | * | * | * |
| Lei, W.Q. et al. | * | - | * | * | * | * | * | * | * |
| Qi, W.X. et al. | * | * | * | * | - | - | * | * | * |
| Feng, J. et al. | * | * | * | * | - | - | * | * | * |
| Liang, X.X. et al. | * | * | * | * | - | - | * | * | * |
| Midik, M.M. et al. | * | * | * | * | - | - | * | * | * |
| Fu, M. et al. | * | * | * | * | - | - | * | * | * |
| Gao, Z. et al. | * | * | * | * | - | - | * | * | * |
| Yekeduz, E. et al. | * | * | * | * | - | * | * | * | * |
| Yang, S. et al. | * | * | * | * | - | - | * | * | * |
| *indicates criterion met; - indicates significant of criterion not met. | | | | | | | | | |
